# Current Status and Future Perspective of Artificial Intelligence in the Management of Peptic Ulcer Bleeding: A Review of Recent Literature

**DOI:** 10.3390/jcm10163527

**Published:** 2021-08-11

**Authors:** Hsu-Heng Yen, Ping-Yu Wu, Mei-Fen Chen, Wen-Chen Lin, Cheng-Lun Tsai, Kang-Ping Lin

**Affiliations:** 1 Division of Gastroenterology, Department of Internal Medicine, Changhua Christian Hospital, Changhua 500, Taiwan; 91646@cch.org.tw; 2 General Education Center, Chienkuo Technology University, Changhua 500, Taiwan; 3 Department of Electrical Engineering, Chung Yuan Christian University, Taoyuan 320, Taiwan; pingyu841215@gmail.com (P.-Y.W.); mei549@gmail.com (M.-F.C.); 4 Artificial Intelligence Development Center, Changhua Christian Hospital, Changhua 500, Taiwan; 5 Technology Translation Center for Medical Device, Chung Yuan Christian University, Taoyuan 320, Taiwan; lin_wenchen@cycu.edu.tw (W.-C.L.); clt@cycu.edu.tw (C.-L.T.); 6 Department of Biomedical Engineering, Chung Yuan Christian University, Taoyuan 320, Taiwan

**Keywords:** peptic ulcer, bleeding, deep learning, artificial intelligence

## Abstract

With the decreasing incidence of peptic ulcer bleeding (PUB) over the past two decades, the clinician experience of managing patients with PUB has also declined, especially for young endoscopists. A patient with PUB management requires collaborative care involving the emergency department, gastroenterologist, radiologist, and surgeon, from initial assessment to hospital discharge. The application of artificial intelligence (AI) methods has remarkably improved people’s lives. In particular, AI systems have shown great potential in many areas of gastroenterology to increase human performance. Colonoscopy polyp detection or diagnosis by an AI system was recently introduced for commercial use to improve endoscopist performance. Although PUB is a longstanding health problem, these newly introduced AI technologies may soon impact endoscopists’ clinical practice by improving the quality of care for these patients. To update the current status of AI application in PUB, we reviewed recent relevant literature and provided future perspectives that are required to integrate such AI tools into real-world practice.

## 1. Introduction

**Figure 1:**
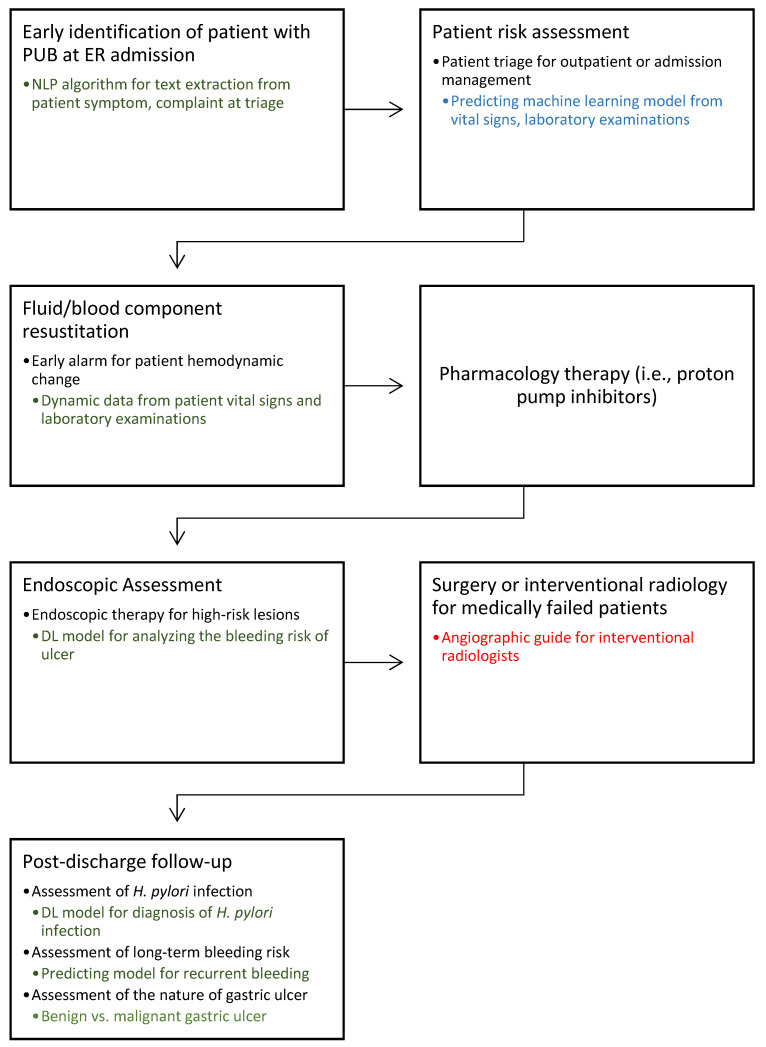
Cascade management of peptic ulcer bleeding. This diagram illustrates the potential role of artificial intelligence (AI) in the future management of peptic ulcer bleeding (PUB) based on text, data, and imaging. Blue: studies with multicenter clinical data validation; Green: studies with single-center clinical data validation; Red: no relevant research found in this field. AI, artificial intelligence; DL, deep learning; ER, emergency room; NLP, natural language processing; PUB, peptic ulcer bleeding.

Peptic ulcer bleeding (PUB) is a common gastrointestinal (GI) emergency requiring prompt assessment, with a mortality rate of 2–10% [1,2,3]. Recently, with the reduced incidence of peptic ulcer disease and the advancement of endoscopic therapy, the bleeding-related hospitalization and mortality rates of PUB have decreased [4,5]. International guidelines have been updating the optimal management approach for patients suffering from PUB [6,7]. The cascade of management can be divided into three stages: pre-endoscopy, endoscopic, and post-endoscopy management. Pre-endoscopy management includes assessing patient’s risk for hospitalization, providing adequate fluid and blood component resuscitation, prescribing medication such as a proton pump inhibitor (PPI), and identifying the timing of endoscopy (Figure 1). Endoscopic management includes assessing the nature of bleeder (e.g., peptic ulcer disease, malignancy, or variceal hemorrhage) and providing endoscopic therapy as appropriate. For post-endoscopy management, intravenous PPI infusion therapy is prescribed to reduce PUB recurrence. Furthermore, eradication of *Helicobacter pylori* infection decreases the recurrence of peptic ulcer disease, and long-term secondary PPIs are required for patients who are at risk for recurrent bleeding.

Developed since the 1950s, artificial intelligence (AI) refers to computer programs that can simulate the human cognitive process in problem-solving and learning [8]. Through the machine learning (ML) approach, the computer can process large data to build various predicting models. Meanwhile, deep learning (DL) has further simulated human neuronal networks with improved performance, especially image processing, since 2010. A UK survey study demonstrated that the gastroenterology trainee experience for PUB management declined from 76% in 1996 to 15% in 2011 [2], owing to the decreased incidence of peptic ulcer disease. The use of AI technology for PUB could enhance the accuracy of patient triage, help achieve accurate therapeutic decisions, and prevent human errors caused by inexperience, especially in an emergency. In this review, we highlight the published literature in the last 5 years with keywords of “artificial intelligence”, “peptic ulcer bleeding”, “nonvariceal bleeding”, “deep learning”, or “machine learning” from a PubMed search to determine the current status and gain insight into the role of AI in PUB management.

## 2. Application of AI in the Pre-Endoscopy Period for Patient Risk Assessment

Upon presentation at the hospital, stratification of patients in terms of gastrointestinal bleeding (GIB) risk is recommended [6,7,9]. Accurately identifying (“phenotype”) patients with GIB during initial assessment is the first step toward patient management, especially during these times of the COVID-19 pandemic. Shung et al. [10] used multiple natural language processing (NLP)-based approaches for automated phenotyping of patients in the emergency department. They found that the syntax-based NLP algorithm from patient triage information performed better than the systematized nomenclature of medicine code information for the patient’s condition, which allows early use of patient triage to subsequent patient management.

In the past two decades, three widely validated scoring systems, namely, Glasgow–Blatchford score (GBS) for outpatient management [11], Rockall score for mortality [12], and the AIMS65 score [13,14,15], have been utilized for predicting low-risk patients. However, compared with these conventional scores [16], ML can potentially improve risk assessment for the need for transfusion, endoscopic evaluation, or hospital admission for observation. Clinical ML use is also more feasible than such conventional scores for busy clinicians through the automatic deployment of ML models with existing available electronic health records in many healthcare systems. In 2003–2008, nine small studies were conducted to investigate ML’s potential for PUB risk assessment in comparison with the conventional scores [16]. The median areas under the curve (AUCs) were higher in artificial neural networks (0.93; range, 0.78–0.98) than in other ML models (0.81, range: 0.40–0.92) when predicting patient mortality, intervention requirement, or rebleeding. Moreover, ML generally provided a better prognostic performance in patients with GIB than conventional scores, and artificial neural networks tended to outperform other ML models.

In 2020, Seo et al. [17] prospectively analyzed 1439 PUB cases to compare the accuracy of ML and conventional scores for PUB patient instability including hypotension, rebleeding, and mortality. Four ML algorithms, namely, logistic regression with regularization, random forest classifier (RF), gradient boosting classifier (GB), and voting classifier (VC), were compared using the GBS and Rockall scores. The RF model was the most accurate in predicting mortality (AUROC: RF 0.917 vs. GBS 0.710), while the VC model was the most accurate for hypotension (VC 0.757 vs. GBS 0.668) and rebleeding within 7 days (VC 0.733 vs. GBS 0.694). The global feature importance analysis identified clinically significant variables, including blood urea nitrogen, albumin, hemoglobin, platelet, prothrombin time, age, and lactate. Thus, the ML models may be helpful in early predicting high-risk patients with initially stable upper GIB upon admission to the emergency department. However, ML performance relies on the quality of data, and these studies usually had a small sample size (<1000 cases) with no external validation data for their performance.

Shung et al. [18] were the first to conduct a large prospective international study for building an ML model for patients with PUB by testing and comparing the performance of the ML model and the conventional scoring system in 2020. They collected patient data from medical centers in four countries (US, Scotland, England, and Denmark; *n* = 1958) to build a model that can predict the need for hospital-based intervention (transfusion or hemostatic intervention) or 1 month mortality. Data from two Asia-Pacific sites (Singapore and New Zealand; *n* = 399) were externally validated. Only nonendoscopic features such as age, sex, clinical symptoms, and laboratory variables (hemoglobin, albumin, international normalized ratio, urea, and creatinine) were selected to build the model. The ML model showed a higher AUC (0.91) than GBS (0.88, *p* = 0.001), Rockall score (0.73, *p* < 0.001), and AIMS65 score (0.78, *p* < 0.001). In the external validation cohort, the ML still achieved a higher AUC (0.90) than GBS (0.87, *p* = 0.004), Rockall score (0.66, *p* < 0.001), and AIMS65 score (0.64 (*p* < 0.001). The proposed ML model improved the identification of low-risk patients who can be safely discharged early from the emergency department. Importantly, this ML model identified more than two times the number of patients with very low risk than the available best-performing clinical risk tool.

After presentation in the hospital, initially stable patients who are at risk for hemodynamic instability requiring blood transfusion must be identified during the dynamic monitoring of the patient status. Levi et al. [19] developed an ML model using publicly available intensive care unit (ICU) databases of 14,620 records with input variables, including several laboratory analyses and demographic information. Their model, which was based on the patient’s vital signs and laboratory test changes in the first 5 h of ICU admission, showed a high level of accuracy (overall AUC, 0.80) in predicting the need for transfusion in the next 24 h of admission.

Therefore, such an algorithm is essential to provide improved risk assessment through the automatic retrieval of information from electronic health records, thereby allowing timely decision support in an already crowded clinical scenario.

## 3. Application of AI during Endoscopy

**Figure 2:**
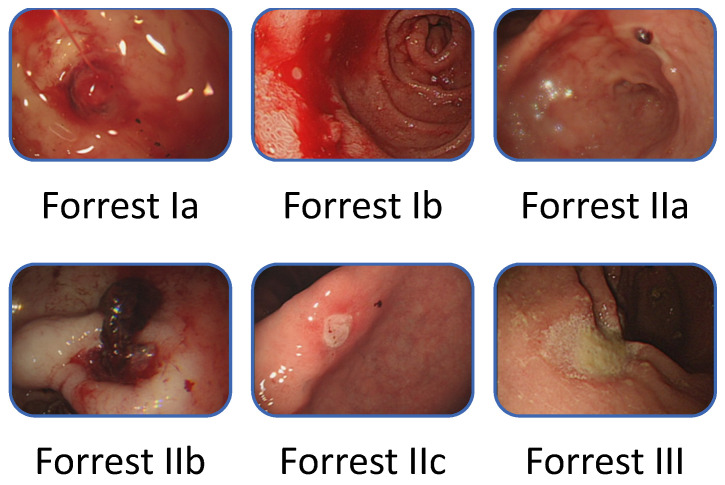
Forrest classification of bleeding peptic ulcers.

Forrest [20] described the endoscopic classification of PUB in 1974 (Figure 2). The classification requires endoscopist judgment of the risk for rebleeding and the need for endoscopic intervention. Current guidelines [3,6,7] suggest that patients who are highly at risk for ulcers, such as those with active spurting, active oozing, or a nonbleeding visible vessel, should receive endoscopic therapy because of the high risk for persistent bleeding or rebleeding, especially when only relying on drug therapy. However, the ability to make a correct classification varies with the endoscopist’s experience, whereby an experienced endoscopist [21,22] can reportedly make better clinical judgment than clinical risk scores [23]. In the study of Laine et al. [24], the rate of correct identification of the endoscopic characteristic of hemorrhage increased as the endoscopic experience increased (performing five cases per month), from 59% to 73% before a training course. After the training course, the increase was related to the training level: fellows, 15% increase; physicians with 0–20 years of experience since training, 8% increase; physicians with an experience of 20 years or more since training, 3% increase. In an Italian study, Forrest Ia/b lesions showed a high interobserver agreement, whereas Forrest II/III lesions exhibited a low agreement [25].

**Figure 3:**
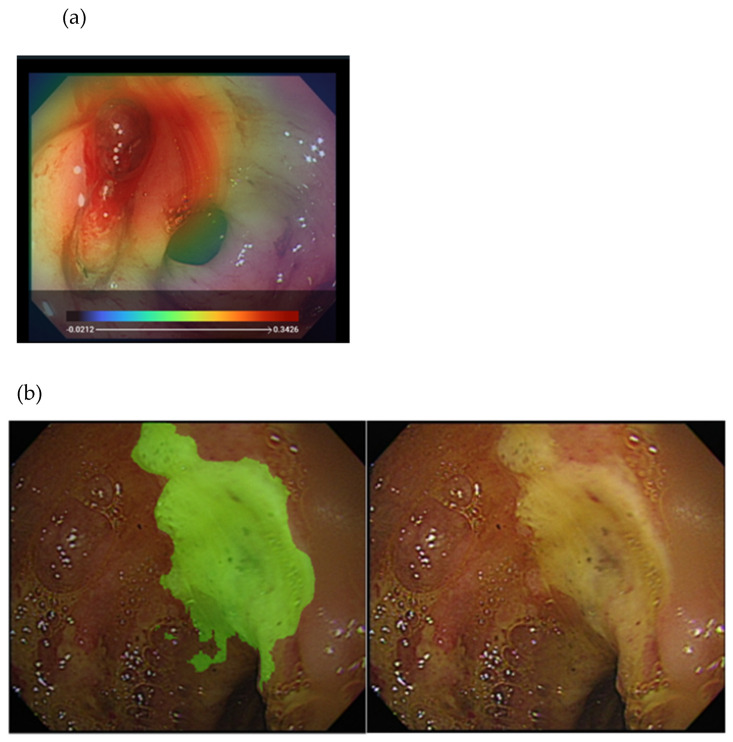
Illustration of the DL approach for analyzing endoscopy images in peptic ulcer disease: (**a**) heatmap image showing an active bleeder in the endoscopy image (**upper**); (**b**) segmentation of the ulcer area (**left**) from the original endoscopy image (**right**).

To explore whether AI is useful for identifying the endoscopic characteristics of hemorrhage during endoscopy, our study [26] initiated the proposal of a DL model that can classify endoscopic images with different bleeding risks according to the Forrest classification and using 2378 still endoscopic images from 1694 patients with PUB (Figure 3). The agreement of the model was moderate to substantial with the senior endoscopist on the testing dataset. The accuracy of the DL model was higher than that of a novice endoscopist. Therefore, the DL model has potential use, particularly in aiding young endoscopists in decision making during emergent endoscopy.

## 4. Application of AI for Patient Care after a Bleeding Episode

Deshmukh et al. [27] proposed an ML model by analyzing 5691 patients with GIB admitted in 2020; patient data were obtained from an ICU database. The performance of the ML model was compared with that of the current standard APACHE risk score. The ML model was more accurate in predicting patient mortality with GIB than the current scoring system, allowing the elimination of low-risk patients from ICU observation. Moreover, combining static and dynamic data could be utilized to build predicting models for long-term patient outcomes after a PUB episode. In 2018, Tan et al. [28] proposed the hybrid residual network (ResNet) and long short-term memory methods, wherein the clinical information between static and dynamic data and temporal dependency information was integrated to improve the accuracy in predicting PUB mortality. Their dataset included 35 types of static variables (e.g., birth date, gender, doses, and medication) and seven types of dynamic time series of laboratory test results. The proposed method achieved an AUC of 0.94 in predicting 10 year mortality in patients after experiencing a PUB episode. Subsequently, they analyzed clinical data from 6367 patients with PUB in Hong Kong to predict mortality; the data included 35 types of static data, such as birthdate, gender, and seven types of irregularly recorded dynamic laboratory parameters (e.g., serial creatinine, hemoglobin, and platelet) [29]. Their model showed an improved prediction ability and provided meaningful interpretations, thereby further helping clinicians in identifying the main risk factors and designing personalized treatments to improve clinical outcomes.

Risk factors leading to PUB must be identified to prevent PUB recurrence after the initial bleeding episode. *H. pylori* infection and the use of nonsteroidal anti-inflammatory drugs (NSAIDs), including low-dose aspirin, are well-recognized risk factors of PUB. Recently, the use of DL has been increasingly evaluated for the computer-aided diagnosis of *H. pylori* during endoscopy. Regarding accuracy, sensitivity, and specificity in detecting *H. pylori* infection during endoscopy, this AI model achieved 87.1% (95% confidence interval (CI) 81.8–91.1), 86.3% (95% CI 80.4–90.6), and 87.1% (95% CI, 80.5–91.7), similar to human endoscopist scores of 82.9% (95% CI, 76.7–87.7), 79.6% (95% CI, 68.1–87.7), and 83.8% (95% CI, 72–91.3), respectively [30].

Eradicating *H. pylori* and avoiding the unnecessary usage of NSAIDs can decrease PUB recurrence following the initial bleeding episode. Patients without an identifiable factor of PUB, otherwise known as idiopathic peptic ulcers, have a poorer prognosis than those with identifiable etiology because of higher rates of PUB recurrence and mortality [31]. Identifying such patients who have a higher risk for PUB recurrence and administering long-term secondary PPIs may improve patient condition [32]. Wong et al. [31] presented a predicting ML model for patients with idiopathic peptic ulcers (IPU-ML model). They retrospectively included 22,854 patients as the training cohort and 1265 patients as the independent validation cohort for predicting recurrent bleeding from idiopathic peptic ulcers using their model. This model incorporated six clinical parameters: age, baseline hemoglobin, and the presence of gastric ulcer, gastrointestinal diseases, malignancies, and infections. It identified patients with recurrent ulcer bleeding within 1 year, with an AUROC of 0.775 and an overall accuracy rate of 84.3%. Clinical application of the IPU-LM model may help clinicians prescribe prophylactics in such high-risk patients and avoid the long-term side-effects of acid-suppressive agents for those at low risk for recurrent bleeding.

As endoscopic biopsy may not performed at the index endoscopy, follow-up endoscopy for these patients is essential because the treatment of benign or malignant gastric ulcers is entirely different [33]. Artificial intelligence systems are being studied for differentiating benign vs. malignant gastric ulcers [34,35,36] and early vs. advanced gastric cancer [35] or for improving endoscopic depth prediction of early cancer [37,38]. Cho et al. [35] developed a convolutional neural network (CNN)-based model to automatically classify gastric neoplasms from endoscopic images. A total of 5017 images were collected for model training, 812 images were used as the test dataset, and an additional 200 images were used for prospective validation. There was no statistical difference between the model and the endoscopist, with the worst performance in the differentiation of gastric cancer (accuracy 76.0% vs. 82.0%). Namikawa et al. [36] reported the improved diagnostic accuracy of the CNN model for classifying gastric cancer or gastric ulcers after adding 4453 gastric ulcer images to the 13,584 gastric cancer images and 373 gastric ulcers in the initial model training. The overall accuracy was significantly improved from 45.9% to 95.9% [36]. Wu et al. [38] developed a deep learning-based AI system covering early gastric cancer diagnosis, cancer invasion depth prediction, and differentiation status. A human–machine competition involving an AI system and 46 expert endoscopists from 19 provinces in China was performed. The system outperformed endoscopists in identifying the cancerous lesions. It was comparable with endoscopists in predicting tumor invasion depth and differentiation status. The application of such AI systems could be a powerful tool to assist endoscopists in the follow-up of patients after the bleeding episode in clinical practice.

## 5. Research Perspective on AI for PUB Management

Although PUB is a common medical emergency, its corresponding patient care remains suboptimal [39] despite the presence of various practice guidelines. The variability of adherence to the practice guidelines has been associated with patient outcomes [40]. Meanwhile, the recent progress of AI has substantially changed people’s lives. Compared with the conventional approach to patient management, applying this technique may further improve our patient care in emergent situations, including PUB, given that it can promptly stratify patient risks, avoid human error, and provide diagnostic assistance.

Although AI models have shown promising roles in managing patients with PUB compared with conventional methods, some limitations have been observed, warranting further investigation. First, the prevalence and etiology of peptic ulcer disease differ between Western and Eastern countries. Most of the advanced AI models from the hospital-based collection of patient data may not be applicable to different ethnic populations. Second, the training quality of the developed model depends on the quality of training data. Public datasets for ML model development exist in many fields of people’s lives [41], but high-quality public health-related information of PUB remains unavailable. Unlike several open datasets that include endoscopy images for colon polyps or capsule endoscopy, no public endoscopy datasets for PUB are available [42,43], probably because of the difficulty in obtaining high-quality endoscopy images during an emergency procedure. As the incidence and prevalence of PUB decrease over time, a prospective collaborative collection of datasets, especially on a national or international scale, is essential to improve the quality and accuracy of model development. Further investigation of the application of AI for real-time diagnostic aid during emergency endoscopy procedures is also required. Third, considering that PUB patient care requires teamwork, the current application of AI in PUB management should mainly focus on the initial patient triage. In addition, a seamless integration of such AI systems with patient electronic medical records and the endoscopy platforms should be considered to improve the current clinical workflow. Fourth, throughout PUB management, interventional radiologists provide an essential salvage therapy position for patients with refractory bleeding [44,45]. Although the application of AI for the analysis during coronary artery angiography has already been researched [46], no such studies have been conducted for GIB cases. Development of such AI assistance tools, especially during a false-negative angiography, is necessary to help inexperienced radiologists identify the culprit bleeder [47]. Fifth, despite the numerous reports showing the similarity or improved accuracy of AI tools compared with conventional methods, a prospective comparative study is needed to show the usefulness and robustness of such AI tools in clinical scenarios to improve patient care quality.

## 6. Conclusions

Patients with PUB, which is a longstanding health problem, may achieve improved care management through a new approach using AI techniques. However, with the declining PUB incidence and clinician experience, further research is needed to apply these techniques in daily treatment practice.

## Data Availability

Not applicable as this review did not report new data.
